# Pit excision with phenolisation of the sinus tract versus radical excision in sacrococcygeal pilonidal sinus disease: study protocol for a single centre randomized controlled trial

**DOI:** 10.1186/s13063-015-0613-5

**Published:** 2015-03-14

**Authors:** Edgar JB Furnée, Paul HP Davids, Apollo Pronk, Niels Smakman

**Affiliations:** Diakonessenhuis, Department of Surgery, Bosboomstraat 1, P.O. Box 80250, 3508 TG Utrecht, Netherlands

**Keywords:** Sacrococcygeal pilonidal sinus disease, Surgery, Local excision, Pit excision, Phenolisation, Randomised controlled trial

## Abstract

**Background:**

Excision of the pit of the sinus with phenolisation of the sinus tract and surgical excision are two treatment modalities for patients with sacrococcygeal pilonidal sinus disease. Phenolisation seems to have advantages over local sinus excision as it is performed under local anaesthesia with a relatively small surgical procedure, less postoperative pain, minor risk of surgical site infection (8.7%), and only a few days being unable to perform normal activity (mean of 2.3 days). The disadvantage may be the higher risk of recurrence (13%) and the necessity to perform a second phenolisation in a subgroup of patients. Wide surgical excision of sacrococcygeal pilonidal sinus disease has a recurrence rate of 4 to 11%. The disadvantages, however, are postoperative pain, high risk of surgical site infection, and a longer period being unable to perform normal activity (mean of 10 days). The objective of this study is to show that excision of the pit of the sinus of sacrococcygeal pilonidal sinus disease with phenolisation of the sinus tract is a successful first-time treatment modality for sacrococcygeal pilonidal sinus disease accompanied by a quicker return to normal daily activity compared to local excision of the sinus.

**Methods/design:**

Patients with sacrococcygeal pilonidal sinus disease will be randomly allocated to excision of the pit of the sinus followed by phenol applications of the sinus tract or radical surgical excision of the sinus. Patients are recruited from a single Dutch teaching, non-university hospital. The primary endpoint is loss of days of normal activity/working days. Secondary endpoints are anatomic recurrence rate, symptomatic recurrence rate, quality of life, surgical site infection, time to wound closure, symptoms related to treatment, pain, usage of pain medication and total treatment time. To demonstrate a reduction of return to normal activity from 7.5 days in the excision group to 4 days in the phenolisation group, with 80% power at 5% alpha, a total sample size of 100 is required.

**Discussion:**

This study is a randomised controlled trial to provide evidence that phenolisation of the sinus tract compared to radical excision reduces the total number of days unable to perform normal activity.

**Trial registration:**

Dutch trial register NTR4043, registered on 24 June 2013.

## Background

Sacrococcygeal pilonidal sinus disease (SPSD) is an acquired disorder of the natal cleft. In patients with SPSD, there are one or more sinus openings in the natal cleft with a blind-ending subcutaneous sinus. It has a prevalence of 8.3% [1]. There is a sex preponderance in men presenting with SPSD between the age of 20 and 30 years [2]. Although 3.7% of patients do not have any symptoms (silent disease), SPSD may cause symptoms interfering with quality of life and social function [1]. Symptoms associated with SPSD are pain, itch and discharge from the sinus with soiling of the underwear. Moreover, infection of the sinus with abscess formation may develop. In patients with silent SPSD, watchful waiting should be the treatment of choice [3]. An abscess requires simple surgical drainage. For patients with complaints due to chronic SPDS interfering with normal daily life, however, several treatment options have emerged in the past years. Simple excision of the pit of the sinus according to Lord and Miller [4], radical excision of the sinus and unroofing of the sinus are frequently used treatment modalities for SPSD [5].

Radical surgical excision of the sinus with primary wound closure or secondary wound healing is the most frequently used treatment for chronic SPDS [4,6,7]. Surgical site infection (SSI), however, is a frequent complication of radical excision with prevalence up to 24% [2]. SSI leads to secondary wound healing which may take several months to cure [8]. The use of a gentamicin-absorbed collagen sponge on the sacrococcygeal fascia reduced the infection rate after primary closure from 20% to 5% in one study [9], although this difference could not be confirmed by another study (26% versus 22%) [10]. The relatively big surgical trauma and the high rate of wound complications after radical excision results in a long wound healing time (mean (standard deviation (SD)) of 15 (4.9) days) and a long mean time to return to normal activity (9.7 (3.6) days) [2,5]. The recurrence rate after excision of SPSD is another problem. Midline wound closure after excision of SPSD has a mean (SD) recurrence rate of 11.1% (0.81%). Off-midline wound closure (Karydakis flap reconstruction), however, possibly seems to decrease the recurrence rate to 4% [2,5].

Another minimal invasive treatment modality for SPSD is excision of the pit(s) of the sinus followed by the applications of phenol into the sinus tract. Phenol has sclerosant properties destroying epithelium and debris in the sinus and is thereby able to promote healing of the sinus [11]. This procedure, first described in 1964, is performed in an ambulatory setting under local anaesthesia [12]. Other advantages of this treatment modality over radical surgical excision of SPSD are a smaller surgical wound with less pain and faster wound healing, and therefore faster recovery and return to normal activity. A disadvantage is the higher recurrence rate. A review of the literature in 2009 [13] reported a mean (SD) recurrence rate of 12.6% (0.91%) after follow-up of 2 years, but without results from randomised controlled trials. Therefore, some patients require another phenolisation treatment to cure the SPSD. The surgical site infection rate after phenolisation treatment was reported as 8.7%. The mean (SD) return to work after the procedure was within 2.3 (3.8) days [13]. Some other cohort studies have been performed since then showing a recurrence rate varying between 8.7 and 33.3% after a follow-up of 22 to 26 months. Mean wound closure time varied from 16 to 28 days, SSI from 0% to 8.7% and return to work from 0 to 3 days [11,14-16].

### Rationale

Excision of the pit of the sinus of SPSD with phenolisation of the sinus tract seems to have advantages over the most used surgical treatment for SPSD - radical sinus excision - as it is performed under local anaesthesia with a relatively small surgical procedure, less postoperative pain, minor risk of SSI (8.7%) and only a few days unable to perform normal activity (mean of 2.3 days). The disadvantage may be the higher risk of recurrence (up to 33%) and phenolisation may have to be repeated a second time. However, high-quality randomised controlled trials comparing both treatment modalities are importantly lacking.

### Objective

The primary objective of this randomised controlled trial is to show that phenolisation of the sinus tract is a successful treatment modality for SPSD and reduces the total number of days unable to perform normal activity.

## Methods/design

This study protocol was constructed according to the SPIRIT guidelines [17].

### Study design

This study is designed as a randomised, non-blinded, single centre, superiority trial with two parallel groups.

### Setting

Patients will be enrolled from a Dutch teaching, non-university hospital.

### Eligibility criteria

#### Inclusion criteria

To be eligible to participate in this study, a subject must meet all of the following criteria: 1) symptoms due to chronic SPSD interfering with daily life; 2) age ≥18 years; and 3) written informed consent is obtained.

#### Exclusion criteria

A potential subject who meets any of the following criteria will be excluded from participating in this study: 1) No or minimal symptoms related to SPSD; 2) suspicion of extensive subcutaneous network of sinus tracts; 3) abscess of SPSD; and 4) previous surgical procedures for SPSD.

### Interventions

#### Intervention: excision of the sinus pit and phenolisation of the sinus tract

Patients are treated in an outpatient setting, under local anaesthesia and aseptic conditions. The patient is positioned in the prone position on the proctology table. The operative area is shaved. No antibiotics are used. The skin is cleaned with an antiseptic solution and covered with a sterile dressing. Circumferential field block infiltration anaesthesia of the sinus is applied, using Lidocaine HCl 2%/epinephrine 1:100,000 (Pharmacist Hospital Haarlem, Haarlem, The Netherlands). Probing via the pit(s) of the sinus is performed to determine the direction of the sinus. Then, a very limited excision of all pits in the midline is made. Additionally, all out-of-midline openings are also excised as little as possible. A small sharp spoon is introduced via all separate orifices to curettage the sinus tract. Attention is paid that all hairs are removed from the sinus tract. After extensive curettage of the sinus tract, accurate haemostasis is reached by external compression. Gauze is used to protect the anus, and the surrounded skin is protected by a coating of vaseline (Pharmachemie BV, Haarlem, The Netherlands). Liquid phenol (85%; Meander Medical Centre, Amersfoort, The Netherlands) is injected by one or more 1 mL syringes (depending on the volume of the sinus tract) with a small catheter via one orifice until phenol is seen at the other orifices. This volume is accepted as the sinus tract volume. The phenol is left in place for 1 minute and aspirated afterwards. This procedure is repeated once. Afterwards, remaining phenol is washed out with ethanol (70%; Fresenius, Schelle, Belgium) to neutralise the phenol. The surrounding skin is cleaned with normal saline. The wound is covered with an absorbing bandage only to prevent soiling of the clothes. The patient is discharged immediately after the procedure.

#### Comparison: radical surgical excision

Patients are treated in a 1-day surgery setting under spinal or general anaesthesia, depending on the preference of the patient or anaesthesiologist. The patient is positioned in the prone position on the operating table. The buttocks are separated with plasters optimising the view of the area of the natal cleft. The operative area is shaved. No antibiotics are used. The skin is cleaned with an antiseptic solution and covered with a sterile dressing. Probing via the pit(s) of the sinus is performed to determine the direction of the sinus. Then, a limited asymmetrical incision of the skin around the sinus is made with the diathermia. An asymmetrical skin incision is preferred to be able to close the wound off-midline. All off- and midline orifices are included in the excision of the sinus. Subsequently, the sinus is radically excised with the diathermia. The subcutaneous tissue is mobilised to be able to close the wound off-midline. Haemostasis is reached by electrocautery. After complete haemostasis, the plasters are loosened to remove the tension on the wound in the natal cleft. A gentamicin-absorbed collagen sponge (Garacol 130 mg sponge; EUSA Pharma (Europe) Ltd, Oxford Science Park, Oxford, UK) is partitioned into numerous small parts and positioned on the sacrococcygeal fascia. Subsequently, the wound is closed off-midline with several separate hand-tied absorbable sutures. The edges of the wound are infiltrated with Lidocaine HCl 2%/epinephrine 1:100,000 (Pharmacist Hospital Haarlem, Haarlem, The Netherlands) for postoperative pain reduction. The skin is closed with separate non-absorbable vertical mattress sutures. The patient is discharged the same day.

### Outcomes

#### Primary endpoint

The primary endpoint is the loss of days of normal activity, measured from the day of operation. Patients do not have any restrictions after the surgical intervention. Complaints due to the intervention are the only factors restricting patients from performing their normal activities. Return to normal activity, such as working or doing housekeeping work, is assessed by filling in a diary during the first 2 weeks after surgery by all participants. As this study mainly includes young and active patients, this outcome measure is highly relevant.

#### Secondary endpoints

The secondary endpoints include symptomatic recurrence rate (assessed by the Visick grading system [18]), symptoms related to treatment (fluid, pain, irritation, itch, burning), usage of pain medication and quality of life. These secondary endpoints are assessed by the patient. Some other secondary endpoints are measured by an assessor with an assessment form including anatomic recurrence of SPSD, time to wound closure (defined as the time from the day of surgery until the day of complete epithelialisation) and surgical site infection.

### Participant timeline

Patients who meet the inclusion criteria will be randomly assigned to either group A (pit excision followed by phenolisation) or group B (primary surgical excision). Patients randomised to group A are treated with excision of the sinus pit and phenolisation of the sinus tract. Afterwards, patients enter into the follow-up programme. If there is recurrent anatomic SPSD combined with symptoms interfering with daily life after at least 6 weeks, the phenolisation procedure is repeated. The treatment in group A can therefore consist of up to two phenolisation sessions. Patients randomised to group B primarily undergo excision of the sinus. Afterward, patients enter into the follow-up programme.

Data regarding quality of life and complaints related to SPSD are obtained preoperatively by questionnaires. Furthermore, the number and locations of pits of the SPSD in the natal cleft are preoperatively assessed. After the procedure, patients enter the follow-up programme. This consists of a diary during the first 2 weeks after the intervention to score complaints related to the surgical intervention, usage of pain medication and loss of days to perform normal activity and, at 1, 2, 6, 12, 26 and 52 weeks after the surgical intervention, quality of life scores, complaints in the natal cleft, satisfaction assessment and wound assessment are measured.

### Sample size

The sample size of this study was calculated based on a reduction of return to normal activity from 7.5 days in the excision group to 4 days in the phenolisation group. A more conservative estimation has been taken into account for both groups as the results shown from the literature are relatively broad (mean (SD), 9.7 (3.6) and 2.3 (3.8) days, respectively) [2,5,13]. The sample size calculation was based on α = 0.05 (two-sided) and a power of 80%. This led to a required sample size of 100 (50 per group) computed by using Microsoft Office Excel 2007 (Microsoft Corporation, Redmond, WA, USA).

### Recruitment

**Figure 1:**
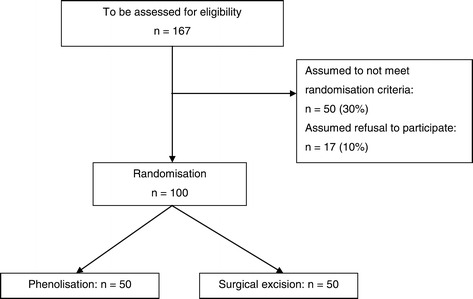
**CONSORT diagram for the study.**

All patients who present to the surgical outpatient clinic of the participating centre with sacrococcygeal pilonidal sinus disease will be considered by the surgeon for participation in this research protocol. Patients with no or minimal symptoms related to SPSD are encouraged to use conservative treatment. However, if conservative treatment is inadequate or symptoms interfere considerably with daily life, there is a reason to proceed to surgical treatment. These patients are assessed if they are eligible for enrolment in this study. With 130 patients presenting with SPSD in the participating centre in 2011 and 2012, an estimation that 30% do not meet the inclusion criteria and assuming a refusal rate of 10%, the inclusion period will be about 2 years (Figure 1).

### Randomisation

**Figure 2:**
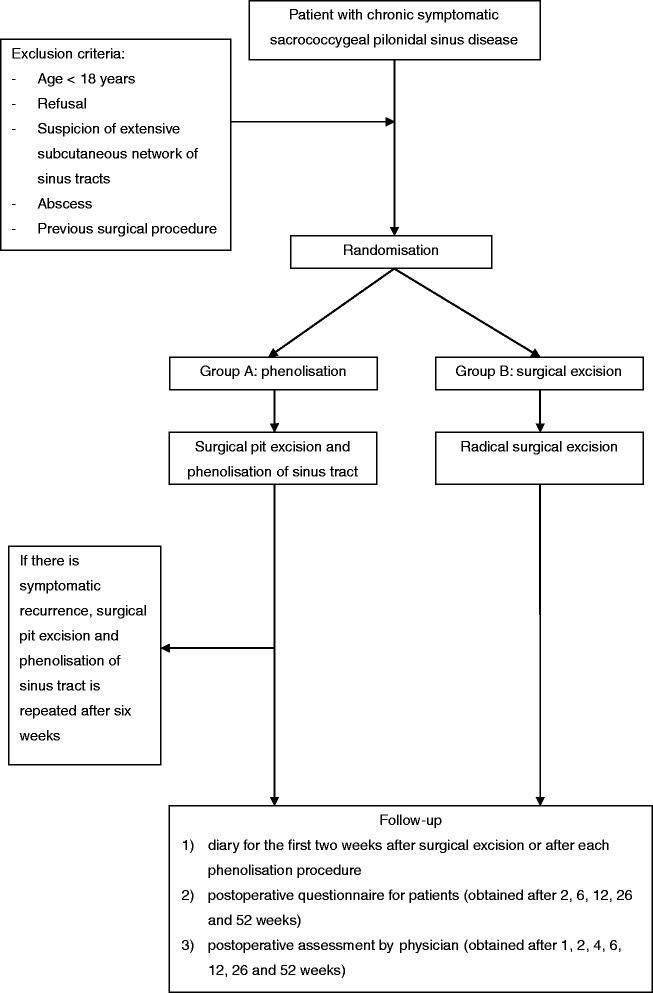
**Study protocol.**

All patients who give consent for participation and who fulfil the other inclusion criteria will be randomised. Patients will be randomly assigned to either group A (pit excision followed by phenolisation) or group B (primary surgical excision) with a 1:1 allocation (Figure 2). The simple randomisation type will be performed by sequentially numbered, sealed and opaque envelopes which will be opened one at a time. Each envelope contains a folded paper with “phenolisation” or “excision” according to the assigned treatment. The content is not visible in any way prior to unsealing and resealing is impossible. These envelopes have been generated by one of the investigator (NS). The assignment schedule is unpredictable and unknown by the principal investigator (EJBF) who will randomise the participants.

### Blinding

Due to the nature of the intervention (that is, because of obvious differences between both interventions) neither participants nor staff members can be blinded to allocation. However, the hypothesis and the primary outcome measure are unknown to the participants.

### Data collection

Preoperatively, complaints related to SPSD (discharge, pain and itch) are evaluated and each symptom is scored by the participants on a six-point scale from 0 (no complaints) to 5 (daily complaints). Quality of life is also preoperatively measured, both by a visual analogue scale (scored from 0 (worst) to 100 (best)) and the Short Form (SF)-36 [19]. The SF-36 is a questionnaire designed to measure health-related quality of life. This questionnaire consists of 36 questions comprising eight different domains of quality of life: physical functioning, physical role limitation, emotional role limitation, bodily pain, vitality, mental health, social functioning, and general health. For each domain, a score between 0 and 100 can be obtained; the higher the score, the better the quality of life.

During the phenolisation procedure, the following items are assessed with an assessment form: number of pit excisions in midline and right and left from the midline and volume of phenol administrated in the sinus. For the excision procedure, the size and depth of the wound is measured as well as the weight of the excision specimen. For both procedures, duration of operation and possible complications are recorded.

A diary is obtained for the first 2 weeks after the intervention to score complaints related to the treatment (fluid, pain, irritation, itch, burning), scored on a six-point scale from 0 (no complaints) to 5 (daily complaints). Additionally, pain is evaluated with a visual analogue scale, scored from 0 (no pain) to 100 (extreme painful), usage of pain medication (yes or no) and if the patient is able to perform normal activity such as working or doing housekeeping work.

The postoperative questionnaire assesses the following items: patient satisfaction by the Visick satisfaction rate (disease scored by the patient as cured, improved, unchanged or worsened; the last two are considered as symptomatic recurrence) [18], symptoms related to the treatment (fluid, pain, irritation, itch, burning; scored from 0 (no complaints) to 5 (daily complaints), and quality of life as obtained by both a visual analogue scale and the SF-36 (both are assessed as described for the preoperative stage).

The wound is postoperatively assessed by an assessor with an assessment form. This form includes anatomic recurrence of SPSD, wound closure (defined as complete epithelisation of the skin) and surgical site infection scored by the Southampton wound scoring system [20]. This scoring system consists of six grades: normal healing (grade 0), normal healing with mild bruising or erythema (grade I), erythema plus other signs of inflammation (grade II), clear or haemoserous discharge (grade III), pus (grade IV) and deep or severe wound infection with or without tissue breakdown (grade V).

To encourage patients to complete follow-up, appointments are scheduled for all patients and a reminder is sent to them 2 to 3 days prior to the appointment.

All forms and questionnaires will be kept in locked cabinets and access to the study data will be restricted. All data will be entered electronically in a SPSS database (SPSS Inc., Chicago, IL, USA) and a password system will be utilized to control access and prevent unauthorised access.

### Statistical methods

The analysis in this study will be performed according to the intention-to-treat principle; that is, all participants, regardless of protocol adherence, will be analysed as randomised [21]. Continuous variable will be summarised using mean (SD) or medians (interquartile range), and binary and categorical data will be summarised using frequencies and percentages, where appropriate. Data will be analysed using SPSS for Windows version 17.0 (SPSS Inc.). We will calculate relative risk and relative risk reduction with corresponding 95% confidence intervals to compare dichotomous variables. The paired-samples *t* test will be used for statistical analysis of continuous pre- and postoperative values and the *t* test for independent samples for statistical analysis of continuous values between both intervention groups. Statistical analysis of categorical values between both groups will be performed using the Pearson Chi-square test, and statistical analysis of categorical pre- and postoperative values by the McNemar test. Differences will be considered statistically significant with *P* < 0.05. We will report reasons for withdrawal for each randomisation group. The effect that any missing data may have on results will be assessed by sensitivity analysis [21,22].

### Harms

Both surgical interventions in this study are generally accepted interventions for SPSD [5,13]. The safety of both procedures has already been established in several previous observational studies and in none of them has a serious adverse event been shown [4,6,7,11,14-16]. During the study any potential side effect or adverse event will be recorded.

### Ethics

This study is conducted in accordance with the principles of the Declaration of Helsinki (59th edition, October 2008) [23]. The study protocol was approved by the local Medical Ethics Committee (United Committees of Human Research, Nieuwegein, the Netherlands; reference number: NL43192.100.13). All patients who meet the inclusion criteria are both orally and in writing informed about the study by their surgeon as well as by the principal researcher. All patients will provide written informed consent before inclusion in the study and randomisation may be allowed. Patients have 1 week to consider their decision whether they will participate in this study. All study-related information will be stored securely in locked cabinets with limited access and password protected databases. Participant study information will not be released outside of the study without the written permission of the participant.

## Discussion

In the past, radical surgical excision was the treatment of choice for SPSD. Since 2010, however, pit excision and phenolisation of the sinus tract has increasingly been applied as treatment for SPSD. This treatment seems to have some advantages compared to radical excision; it is performed under local anaesthesia with a relatively small surgical wound, less postoperative pain and minor risk of complications of the wound (that is, SSI) and long-lasting wound healing. All these advantages potentially lead to a reduction in days unable to perform normal activity. The recurrence rate is, at 13%, somewhat higher for the phenolisation technique. However, as far as we know it seems unlikely that this treatment does compromise or negatively influence radical surgical excision if necessary in the future due to recurrence after phenolisation. Currently, no randomised controlled trials have been performed comparing both treatment modalities. Therefore, the abovementioned advantages of the phenolisation technique over radical excision are not evidence-based. The study described in this research protocol is potentially able to provide evidence of the advantages of the phenolisation technique.

The primary endpoint in this study is the number of days unable to perform patient’s normal activity. This was chosen as all the advantages of the phenolisation technique (that is, local anaesthesia and less pain and wound complication) contribute to this primary endpoint. As stated in the research protocol, a more conservative estimation of the primary endpoint was considered for sample size calculation, as the results from the previous observational studies showed a relatively broad SD [2,5,13]. Moreover, this more conservative estimation requires the inclusion of more patients into each study group, leading to the advantage of a more powerful randomised trial. The sample size could also be calculated based on the difference in recurrence rate between both interventions. Although the recurrence rate of surgical excision seems to be favourable, the difference with the phenolisation technique is quite small as reported in previous studies [5,13]. Therefore, it would require an inclusion of over 4,000 patients in each group to reach statistical significance. It is, in our opinion, unattainable to perform such a randomised controlled trial.

The most important disadvantage of the phenolisation technique is the somewhat higher recurrence rate. An extensive subcutaneous network of sinus tracts may result in treatment failure when phenol does not reach all the tracts [16]. This may contribute to recurrence. Therefore patients with a suspected extensive network of subcutaneous tracts are not considered for inclusion in this study protocol. The traditional treatment consisting of surgical excision should be the treatment of choice in these patients. Suspicion of an extensive subcutaneous network should especially be raised if more than three off-midline orifices are present. Another factor that may lead to recurrence after phenolisation is inadequate haemostasis after debridement as this prevents sufficient contact between the walls of the sinus tracts and phenol. Additionally, an essential step of the phenolisation technique required to reduce the recurrence rate is removal of all hairs from the sinus tract(s) as these are not destroyed by phenol. All these items may result in recurrence, and therefore repetition of treatment with the phenolisation technique may be required in a subgroup of patients. These three essential steps in the phenolisation procedure have been incorporated in the current study protocol to confine the recurrence rate to a minimum. A second treatment with phenolisation, however, will be inevitably necessary in a subgroup of patients. A second procedure is included in the phenolisation group (group A) in this study, if required. However, a third phenolisation session is not performed in this study in the case of recurrent SPSD after a second time of phenolisation, as Dag and colleagues reported a failure rate of 98% in patients with three or more phenolisation sessions [11].

In conclusion, this study is a randomised controlled trial to provide evidence that sinus pit excision followed by phenolisation of the sinus tract compared to surgical excision reduces the total number of days unable to perform normal activity.

## Trial status

The first patient was randomized on 20 September 2013. To date, 40 patients have been included in the study. The end date of this study is currently expected to be February 2016. This study is registered in the Dutch Trial Register (NTR4043).

## Abbreviations

SD: Standard deviation
SF-36: Short-Form-36
SPSD: Sacrococcygeal pilonidal sinus disease
SSI: Surgical site infection
